# Navigating the First Months of Work: A Cross-Sectional Study on Newly Graduated Registered Nurses' Journey Into the Profession

**DOI:** 10.1155/nrp/9555767

**Published:** 2025-10-10

**Authors:** Annika Eklund, Maria Skyvell Nilsson, Agnes Olander, Anders Sterner

**Affiliations:** ^1^Department of Social and Behavioural Studies, University West, Trollhättan 46186, Sweden; ^2^Department of Health Sciences, University West, Trollhättan 46186, Sweden; ^3^Faculty of Health Science, Kristianstad University, Kristianstad, Sweden; ^4^Faculty of Caring Sciences, Work Life and Social Welfare, University of Borås, Borås 50190, Sweden

## Abstract

The transition process for newly graduated registered nurses (NGN) entering hospital work is critical in managing challenges like insecurity and stress, while also supporting role navigation, learning, job satisfaction and retention. This study aimed to explore NGNs' perceptions and experiences during their first four months transitioning to hospital work. A cross-sectional survey with Likert-scale questions and two open-ended free text questions was conducted with NGNs enrolled in a regional transition program at five Swedish hospitals, gathering data from 216 nurses between September 2021 and September 2022. Descriptive statistics and qualitative content analysis were used to assess the data. The findings revealed that 61% of NGNs felt well prepared by their undergraduate nursing education, and 85% enjoyed their profession as nurses. Most reported they were integrated into their teams, satisfied with their support, and understood their responsibilities. However, only 28% reported feeling fully recovered for work, and 44% expressed fear of making mistakes. Qualitative responses highlighted feelings of unpreparedness for the workload, pace and ward-specific routines, although opportunities for learning, building relationships and validating their skills were viewed positively. The transition experience was influenced by individual, social, and organisational factors. Ongoing improvement in transition processes requires shared responsibility between nurses, educational institutions and hospitals.

## 1. Introduction

This study contributes to the existing literature by addressing the critical early phase of newly registered graduated nurses' (NGNs) transition into clinical practice, specifically the first four months. This phase has been underexplored despite its significance for clinical skills, role clarity and managing insecurity and stress [1, 2] and influencing their decision to remain in the profession [3]. Studies have also reported an increase in competence between months 0–3 in the profession [4], indicating a value to highlight and further explore this phase. While many studies have explored the first year of practice and transition programs [5, 6], there is a need for a more comprehensive understanding of nurses' preparedness for work, challenges and rewarding aspects encountered during the first months in the profession. Such knowledge is crucial for both educational institutions and healthcare organisations to design targeted support strategies that enhance retention and professional development.

## 2. Background

Transitioning from being a nurse student to becoming a clinical practitioner is a significant step that requires personal capability and confidence [7], as well as a supportive learning context [8]. This process has been reported to cause a range of stressors as expectations, work practices and values tend to conflict with realities of workplaces, with heavy workloads and lack of support [9–11]. A recent systematic review by See et al. [12] found that NGNs experience work overload, leading to mental and physical exhaustion. Previous research provides important insights into how NGNs perceive their preparedness for clinical practice. For example, one study found that 65% of NGNs who had just begun clinical work felt their nursing education had adequately prepared them for general nursing duties, and many still reported fear of making mistakes, a demanding workload and lack of confidence in providing nursing care [13]. These challenges are compounded by the need to perform complex tasks they are not fully prepared for, making job demands a significant source of stress [14]. In addition to these individual struggles, NGNs' vulnerability during transition increases the risk of errors [15], further amplifying workplace pressure.

The challenges for NGNs entering clinical work were recognised early on, including experiencing a “reality shock” [16]. Shock encompasses here the individual's social, physical and emotional reactions when confronting new roles and unforeseen or adverse events in a new environment, often related to experiencing a mismatch between perceived readiness and actual clinical competencies [17]. Building on this, Duchscher [18] described the transition as a staged process in which NGNs gradually adapt to their professional roles over the first year. During the initial 3-4 months, nurses primarily focus on “doing”—that is, learning procedures, performing tasks, seeking acceptance within their teams and managing the balance between work and personal life [18]. Around the four-month mark, progressing towards a “being” phase, they typically gain more confidence in their responsibilities but may also experience lingering insecurity in more complex areas such as leadership. This leads them to seek familiarity and predictability in their practice. Next, the “knowing” phase marks the nurse's shift from task-focused performance begins to understand the why behind nursing actions [18]. At this stage, between 6–12 months of work, the nurse develops deeper clinical reasoning, connects theory with practice and gains confidence in making judgments based on their knowledge rather than rote procedure.

However, a review of Graf et al. [19] suggested that the progression through transition phases is not always linear. A lack of structured support, high workloads or insufficient preparation was reported to prolong the “doing” phase, delaying the development of professional confidence and identity. On the contrary, positive interactions with colleagues, a supportive work environment and structured transition programmes have been shown to ease the transition process [7, 20, 21]. More specifically, social support from colleagues and managers has been reported as crucial in reducing work stress, improving care quality and strengthening professional commitment [3]. Such findings are particularly significant because research suggests that transition experiences directly impact retention both in the workplace and the profession [7, 22].

The importance of collaboration between educational institutions and workplaces to ease the transition shock should not be overlooked. Baharum et al. [23] argue that a successful transition depends on contributions from both academic and clinical settings, as well as the individual NGNs themselves. Furthermore, NGNs satisfaction with nursing education has been linked to higher job satisfaction and perceived care quality [24]. Evidence also suggests that NGNs who feel capable of mastering workplace challenges are less likely to leave the profession [25, 26]. In this context, higher readiness for practice, which is shaped by nursing students' learning experiences, has been shown to reduce transition shock and turnover intentions after one year [22].

Without ranking or grading the facilitators described above, it is evident that the initial stages of transition of NGNs are a particularly complex event. Therefore, Hampton et al. [27] argued that it is imperative for hospital organisations to expand their focus beyond clinical aspects and tackle nonclinical subjects like socialisation and stress management, which are known to follow early transition. Given the significance of the transition experience for both individual nurses [19], and the health care system, it is essential to explore and evaluate the experiences of NGNs beyond the first year in clinical practice and transition programmes [28], and beyond technical skills development [6]. Greater attention should be given to nurses' experiences after the initial 3-4 months, as these experiences can impact their well-being, readiness for the next stage of their transition and intention to stay. To contribute to this research field, the aim of this study was to explore perceptions and experiences of NGNs' first four months of transitioning to hospital work. More specifically, NGNs' perceived preparedness, challenges, rewarding experiences and satisfaction with their nursing profession were examined.

## 3. Method

### 3.1. Design

This study was designed as a cross-sectional descriptive survey study. The survey incorporated both qualitative and quantitative data, both of which were considered equally important in interpreting the study's aim [29]. The web-based survey comprised five-point Likert-scale questions (scale ranges: *strongly disagree*, *disagree*, *neither agree nor disagree*, *agree* and *strongly agree*) and two open-ended free-text questions. It also included demographic questions regarding age, biological sex and previous healthcare work experience. Six items focused on participants' experiences of their undergraduate nursing education and satisfaction with their chosen profession and employment. Eight items explored aspects of their workload, support and role clarity. Timeframes were tailored to the nature of each construct. One free-text question requested experienced challenges the last two weeks. Specific recall periods (e.g., past week) were used to reduce recall bias for fluctuating experiences [30, 31] such as workload and support. A broader timeframe (e.g., first months) in the free-text question regarding rewarding aspects allowed the respondents to reflect on their general learning and adaptation process, not just recent events.

To ensure content validity, the researchers developed the survey in collaboration with the steering group for the regional transition program. For improved face validity, two focus groups (*n* = 8) of NGNs participating in the transition program completed the survey. They completed the survey individually and then discussed their impressions of the questions. Their feedback helped identify inconsistencies or specific questions requiring revision or clarification. The research team reviewed this feedback and made final adjustments to the survey. The eight surveys from the focus groups were not included in the final sample of this study.

### 3.2. Setting and Data Collection

The study was conducted in a southwestern region in Sweden with one university hospital and four county hospital administrations. In total, approximately 475–560 NGNs are employed at the hospitals each year, although the exact number varies annually and more are employed during the fall. All NGNs enrolled in a 1-year regional transition program were eligible to participate. NGNs were registered nurses (RN) with a bachelor's degree in nursing. At the time of participation, they had four to seven months of work experience as RNs.

The transition program commences twice a year and has been obligatory since 2018 for all new graduates with less than four months of working experience as RNs. The coordination of the program is overseen by a regional steering group, comprising one appointed representative from each hospital administration. The unit managers in each ward are responsible for the registration of the newly employed NGNs into the program. The program consists of introduction and supervision at the ward, lectures, simulations and group reflection seminars with other NGNs, to build competence, confidence and professional belonging. Although the program is jointly coordinated, the hospitals retain the flexibility to adapt certain components to meet local needs or conditions. These adaptations may include specific lecture content or scheduling and format of reflection seminars.

Data were collected anonymously via a web-based survey with three different groups of NGNs starting the program in autumn 2021, spring 2022 and autumn 2022. The research team had no direct access or connection to the participants at any point of the study. Instead, representatives from the transition program at each hospital sent out written information prepared by the research group. This information included details about the study, its objectives, methods for data storage and analysis, researcher contact details and a web-link to the online survey for prospective participants. It was not possible to submit the survey without answering all the questions.

### 3.3. Quantitative Data Analysis

The study utilised IBM SPSS Statistics Version 28 [32] for analysis of quantitative data. Descriptive statistics, including percentages, means, medians and standard deviations, were computed. Additionally, frequency analyses were performed to identify any possible missing values or errors, and outline demographic characteristics. For analysis of the Likert scale questions, responses were grouped, with “*agree*” and “*strongly agree*” classified as agreement, while “*strongly disagree*,” “*disagree*” and “*neither agree nor disagree*” were considered disagreement. Associations between demographic variables and ordinal data (age were categorised as “younger” and “older” using median as cutoff) were analysed using chi-square tests, with a two-tailed *p* value of < 0.05 regarded as statistically significant.

### 3.4. Qualitative Data Analysis

The free text responses were analysed using qualitative content analysis [33] and NVivo 14 Plus Software [34] to support data sorting. The qualitative data included participants' free-text responses to the following open-ended questions: (1) What has been your biggest challenge at work this past week? and (2) What has been most rewarding at work in your first months in the profession?

Since these questions pertained to different contexts and timeframes, they were analysed as distinct units and presented individually. However, the analysis of the questions was performed in similar ways. The initial step was to read through all the free text responses and make initial notes about the character and content of the statements. The statements were mostly relatively short, and consequently, a more manifest interpretation—that is, to focus on the visible and obvious components in the text [33]—was considered appropriate. The analysis was conducted in several steps. Firstly, all meaning units—that is, the pattern of words or statements related to the same fundamental meaning—were coded (Tables 1 and 2). In the next step, all codes, and associated meaning units sharing a communality of content, were sorted into tentative categories. The final step involved a comprehensive discussion of how these reflect the study's findings, which resulted in the final categories that are used to describe challenges and rewarding experiences.

## 4. Results

### 4.1. Quantitative Analysis of Survey Responses

A total of 216 nurses chose to participate by responding to the survey. Of the respondents, 87% were women, the mean age was 29.5 years, and 77% had previous working experience from health care before being employed as RNs (primarily as assistant nurses at hospitals or home care services). Characteristics of the respondents are presented in detail in Table 3.

A chi-square analysis was used to investigate the relationships between demographic data and scores (dichotomised into agree or disagree as shown in Table 4). Older nurses, with median age as cutoff, were significantly associated with plans to continue working as a nurse *χ*^2^ (1, *N* = 213) = 6.565, *p*=0.010. The analysis also showed that older nurses were significantly associated with the idea to choose nursing education if they were to choose an education programme again *χ*^2^ (1, *N* = 213) = 8.249, *p*=0.004. Biological sex or work experience was not found to be statistically significantly associated with any of the questions analysed.

A range of questions about nurses' present situation on the ward and on experiences during their last week were analysed. A high score indicating perceptions of a higher workload yielded a mean score of 3.97 (SD 0.826), indicating a high or very high workload for 72% of the nurses. Furthermore, 28% of the nurses perceived themselves as having recovered before starting a new shift often or very often during the last week. During the last week, 44% of the nurses had often or very often worried about making mistakes. At the same time, an overwhelming majority of nurses often felt that they knew what responsibilities they have on the care unit (90%) and felt like a part of the team on the care unit (90%). These additional analyses are presented in Table 5.

A chi-square analysis was used to investigate the relationships between demographic data and scores (see Table 5). No statistically significant associations were found between age, biological sex or work experience and any of the analysed questions.

### 4.2. Qualitative Analysis of Responses to Free-Text Questions

Of the participants, 170 chose to respond to these questions. The free text answers to the first question were between one (e.g., “stress” and “workload”) and 64 words, with a total of 1421 words. The free text response to question two was between one (e.g., “patients”, “colleagues” or “everything”) and 59 words, with a total of 1364 words.

This section presents the analysis of the free-text responses regarding the NGNs experienced challenges in the past 2 weeks and the experienced rewarding aspects of their first months of work (see Table 1).

#### 4.2.1. Experienced Challenges

The analysis of experienced challenges the past 2 weeks reported by the NGNs were categorised as “pace and complexity of work”, “emotions and self-imposed expectations”, “lack of recovery” and “insufficient knowledge and learning opportunities,” which is elaborated on below.

##### 4.2.1.1. Pace and Complexity of Work

The high workload and the number of patients to be responsible for were highlighted as very challenging for the NGNs. This was caused by staff shortages, overcrowded wards, high patient flow and completing assignments quickly. They also described challenges related to quickly adjust to novel tasks and situations:

“*I had to quickly switch to a specific task I had never done before, there were few colleagues around who had done the same.*” (R74); “*The high patient flow, overwhelmed with tasks, impossible to manage that stress*” (R69).

The pace of work was repeatedly considered too fast in relation to the experience the NGNs possess. More specifically, the challenges related to having time for everything that needed to be done, and to not being ready to take responsibility for situations when everything must be solved quickly. They also described roles and tasks that were particularly challenging, such as being a section leader, or coordinating the discharge of patients, administering drugs they were previously unfamiliar with or dealing with language barriers.

Further, the NGNs described that it had been challenging to handle new and complex patient and family situations, deteriorated patients, and to perform complex medical tasks:

“*Acting and making decisions in emergency situations. When quick decisions need to be made, I don't feel knowledgeable enough*” (R196); “*Difficult when a patient gets very sick, don't really know what to do*” (R100).

Related to complexity, the requirement to be able to plan work and prioritise correctly among many patients was emphasised as highly challenging: “*Prioritise the tasks correctly*” (R170); “*Many patients with a large burden of care at the same time, feel that I need to be more experienced and have more knowledge in my field to be comfortable …*” (R24).

##### 4.2.1.2. Emotions and Self-Imposed Expectations

The nurses expressed different aspects reflecting the feeling of “being new” as particularly challenging: too much responsibility, feeling insecure, the feeling of having chosen the wrong profession, ethical stress and dilemmas that affect work or the inability to let go of control.

They also expressed a lack of self-confidence in relation to their own knowledge, competence and abilities: “*To trust myself, that I can and do the right thing*.” (R220).

Several nurses believed that the most challenging experience in recent weeks was the feeling of not being enough for patients, relatives and colleagues. Above all, the lack of time for patients who need support was highlighted:

“*Can't do that little extra, take the slightly longer conversation with the patients when you clearly see that they need it.*” (R275)

##### 4.2.1.3. Lack of Recovery

The NGNs described how they had found it hard to recover over the past few weeks, as their daily work involved an excessive amount of work, extra shifts and a stressful job that causes them to experience constant fatigue. Limited opportunities to take breaks at work hinder proper rest. Some also described difficulties letting go of work after working hours and sleeping well enough.

*“The stress, I'm constantly tired and feel that I never get enough rest.”* (R26)

This category also reflected challenges related to work–life balance. The nurses described examples from their private lives that have burdened them and thus affected the possibility of recovery, such as taking care of relatives.

##### 4.2.1.4. Insufficient Knowledge and Learning Opportunities

Several nurses described the challenge of being able to learn and develop at work in recent weeks. This largely reflected the specific tasks and work organisation in their respective wards: *“To get all the routines in”* (R109).

It was expressed as challenging to experience knowledge gaps regarding specific medical knowledge, organisation of work and routines in, for example, care transitions:

“*Understanding the chain of care, who to contact about what. Which work category/person is responsible for contacts outside the health care system*” (R75).

They also described a lack of opportunities and energy to learn. For example, they describe difficulties in *“Remembering what you think and sorting your thoughts”* (R223), and translating theoretical knowledge to practice.

#### 4.2.2. Rewarding Experiences

Analysis of the participants reported most rewarding experiences during the first 4 months of work was categorised as “opportunities for developing knowledge,” “interacting and building relationships with colleagues and patients,” “being capable of managing nursing duties” and “experiencing meaningfulness of work,” which will be elaborated below (see Table 2).

##### 4.2.2.1. Opportunities for Developing Knowledge

The nurses described their work as highly rewarding, with opportunities for both professional and personal growth, expressed as *“To learn what the profession of a nurse is really like”* (R276); “*Tasks that I have never done before but find solutions to together with colleagues*” (R40). The NGNs expressed several specific aspects as rewarding for continuous learning in their daily work, getting opportunities to meet different types of patient situations and being able to immerse oneself in how the care works at the unit and in the health care organisation.

It was also expressed as rewarding to have opportunities to develop new and in-depth knowledge in both medicine and nursing, but also to develop knowledge of routines and how other professions work. Several respondents highlighted supervision as particularly rewarding in learning situations, with opportunities to develop together with more experienced colleagues. The feeling of “*standing on your own two feet and learning everything for real*” (R259) as a confirmation of their own competence was rewarding.

##### 4.2.2.2. Interacting and Building Relationships With Colleagues and Patients

The nurses described the interaction and developing relationships with colleagues and patients as very rewarding during the first months in the profession. Among their comments were *“Meeting patients and wonderful colleagues”* (R158) and *“The most rewarding thing has been my colleagues*!” (R281). More specifically, colleagues available for questions and being patient and receptive were expressed as particularly important. Also, group supervision with other NGNs, as an activity of the transition programme, was mentioned by several as valuable for building relationships and growing professionally.

The nurses also described patient contacts as very rewarding: “*Nice meetings with patients. I now feel safe*” (R151); “*When you start to feel trust from patients*” (R69).

##### 4.2.2.3. Being Capable of Managing Nursing Duties

Being able to do the job as a nurse, to coordinate and lead the nursing work were described as rewarding aspects. Managing practical and emergency situations in collaboration with others, as well as being able to handle professional demands of work, were emphasised as important. The nurses emphasised the feeling of being able and taking personal responsibility as important: *“To see and perform many tasks and work independently”* (R216). Several even described that the most rewarding thing has been *“everything in the job”* (R71); *“To be able to work practically with what a nurse should be able to do”* (R271).

The feeling of being able to do more than they thought and the opportunity to take own initiatives and responsibility and grow into the role were emphasised. This experience was also expressed as having chosen the right profession: “*The feeling that I am in the right place*” (R95).

##### 4.2.2.4. Experiencing Meaningfulness of Work

The NGNs considered the ability to contribute to patients' health improvement as highly rewarding: “*To help people who are in need*” (R272). Being able to make a difference for patients, feel appreciated by patients, experience their trust and feel that they are doing good were valued.

In addition, being able to contribute knowledge to healthcare, make suggestions and participate in discussions about patients' care was described as rewarding. Being asked by colleagues and other staff about the care work gives a confirming feeling of being needed.

## 5. Discussion

This study aimed to explore perceptions and experiences of NGNs' first four months of transitioning to hospital work. The analysis revealed that a large proportion of nurses felt that their education did not adequately prepare them for practical nursing work, and a high workload, fear of making mistakes and lack of recovery were significant challenges for many NGNs. Despite these stressors, most respondents reported satisfaction with having chosen the nursing profession and intended to stay. They also found their work rewarding, particularly in terms of professional growth, collegial support and the meaningfulness of their contributions to patient care.

Only 57% of NGNs reported that they perceived their nursing education as sufficiently preparing them for practical nursing work. The qualitative data suggested that this reflects a lack of preparation for both the workload and the highly specific and complex nursing tasks they are expected to handle on the wards. A possible explanation to this finding was that the NGNs are in the “doing” phase, where they are occupied with performing tasks and adjusting to practice [18]. Still, this perception of inadequate preparation is not a novel finding—similar concerns have been highlighted in earlier studies—the gap between theoretical education and clinical practice has been shown to create frustration among NGNs, which potentially hinders their integration into the professional role and impedes their long-term professional development [35–37] and impacts turnover intentions during transition [22]. This highlights the crucial need for collaboration and clear structures for responsibilities between educational institutions and hospitals for supporting readiness for clinical practice.

Graf et al. [19] argue that NGNs transition into the workforce with limited clinical training, which often cause a gap between theory and clinical practice which in turn could extend the possibility and duration of transition shock. However, European Union [38] directives have recently mandated an increase in practical training within undergraduate nursing education. These directives aim to better align educational content with the realities of nursing practice by integrating more clinical hours and hands-on experience during the training period [38], but it will take time before any consequences on practical and theoretical preparedness will be observable. However, previous research has emphasised that providing time and opportunities to apply theoretical knowledge in practical settings facilitates the development of essential clinical skills and fosters a deeper understanding of professional responsibilities [39, 40]. Still, in the present study, 90% of the NGNs reported that they know what their responsibilities are at their unit. This might reflect the relatively high percentage (72%) that reported that their undergraduate nursing education prepared them well for theoretical aspects of nursing. Another interpretation would be that the care units have clear and adequate introduction and responsibility structures.

However, 44% of the participants in this study reported fears of making mistakes, which might reflect both the high workload and difficulties prioritising when dealing with urgent situations. Such fears and frustration could also be related to the doubts with their own competence during the first months of work that is inherent in the phenomenon of being new [18]. Being insufficiently prepared and high workload has previously also been highlighted as prolonging the “doing” stage [18, 19]. Still, although the NGNs in this study reported being insufficiently prepared for practical nursing work, solving challenging tasks and being able to work independently was expressed as a validation of their competence and contributing to growth in confidence. This indicated that, rather than reducing challenging tasks and situations such as acute care, the importance is structured learning opportunities and the availability of daily support and supervision to be able to develop required competences during the first months [4, 41]. Being confident in making decisions and mastering nursing tasks safely has been pointed out as important for the NGNs' confidence and to remain engaged during the “being” stage of transition [18, 26]. Further, the NGNs described that it has been challenging to handle issues such as complex patient situations and deteriorated patients. Here, simulations have been reported as a resource for novice nurses to develop abilities to provide care in acute situations, but also to confirm the skills that they possess [42].

Although not explicitly highlighted in transition theory, recovery is crucial for emotional balance and to prevent stress-related illness during the NGNs first months of work [1]. Only 28% of respondents in this study stated that they often or very often feel recovered when starting their job shift. The analysis of the qualitative responses indicated that this could be related to the experienced high workload and fast-paced work, which are well-known aspects of work environmental demands that hampers learning [9] and increase intention to leave [26]. In the present study, NGNs reported challenges in detaching from work, in terms of carrying work-related stress into their personal lives. The findings also align with [2], who identified burnout and inadequate recovery time as critical issues. This lack of recovery not only exacerbates fatigue but also undermines their professional performance and overall effectiveness. These findings shed further light on not only focus on the individual and professional nurse development in transition programmes but also initiative on organisational level to support job-satisfaction and a healthy work environment [43]. Workplaces where challenges such as shock, stress and burnout among NGNs are considered shared concerns are most successful in retaining staff [44]. For example, adopting a holistic approach to workflows and schedules that prioritise rest helps address personal challenges and minimise the risk of prolonged stress [2].

Despite the experienced challenges, 85% enjoyed their profession as a nurse and 84% plan to stay in the profession. It should be noted that being an older nurse, using median as cut-off (28 years), was associated higher intentions to stay and satisfaction with their choice of education. Previous studies have found that younger NGNs report higher dissatisfaction with the profession and their tasks, while older NGNs may prioritise stability [3]. Further, 90% of the participants agreed that they had someone to ask if they need help and felt as a part of the working group, which is notable since socialisation and relational factors are important resources for job satisfaction and intention to stay [1, 23, 26]. NGNs in the present study emphasised the value of available support and guidance from preceptors and experienced colleagues. Besides mitigating the major stressor of transition shock [41], proper support can be a key to bridge the gap between theory and practice and reduce emotional overwhelm [19] and to become more confident in handling clinical skills [7, 45]. The participants also emphasised the value of structured team-based support mechanisms, such as group supervision, which facilitated reflection, shared learning and emotional resilience. These mechanisms have also been shown to ease the transition [2].

### 5.1. Methodological Considerations

One limitation of this study was the unknown response rate, as the number of NGNs who received the survey is not known. The web link to the survey was distributed by programme coordinators at the hospitals, either through email or by writing the web address on a whiteboard. In some cases, the link was not shared at all due to miscommunications or concerns about adding extra stress for the NGNs during the already challenging transition into the workforce during the COVID-19 pandemic [46]. Further, no statistically significant associations were found when using biological sex as a variable. This should be interpreted with caution as it may be due to low representation of males in the study (12% of the participants). Regarding the qualitative analysis, the free-text responses were typically brief, and because respondents were anonymous, we could not ask for clarifications or follow-up responses. While the open-ended survey responses limited the exploration of deeper underlying meanings and comprehensive analysis, they still offered valuable illustrative insights of common themes while preserving participants' own words that complemented the quantitative data.

## 6. Conclusions and Implications

The results of this study showed that a large proportion of NGNs, after four months of hospital work, felt that their nursing education insufficiently prepared them for nursing work. The analysis indicated that the initial journey into the profession involved both professional and personal challenges and growth. In addition, the results demonstrated that both challenging and rewarding aspects of transition that the NGNs report represent organisational (workload), relational (building relationships) and individual aspects (ethical stress and self-imposed expectations). Studies exploring NGNs experiences after one year in the profession have previously been reported similar factors as important for learning and retention. This means that these aspects should be carefully considered when organising work and targeting interventions to support learning, socialisation and job satisfaction already during the first months of work to ease the transition and to increase retention. Another implication of this study was that transition programmes and lectures only might not cover the specific challenges and need for support that the NGNs express; those are questions for the general working conditions and support structures at the wards.

## Figures and Tables

**Table 1:** The most demanding experiences for newly graduated nurses during the past 2 weeks, including number of statements for each category.

Categories (number of statements)	Codes
Pace and complexity of work (*n* = 110)	Workload
	Work pace task management
	Handling coworkers

Emotions and self-imposed expectations (*n* = 30)	Being new
	Responsibility
	Lack of security and confidence
	Ethical stress
	To release control
	Absence of time for patients and coworkers

Lack of recovery (*n* = 15)	Recovery
	Social life
	Stress

Insufficient knowledge and learning opportunities (*n* = 9)	Knowledge gaps
	Lack of time for learning
	Energy for learning

Don't know (*n* = 4)	

**Table 2:** The most rewarding experiences for newly graduated nurses during their first months in the profession.

Categories (number of statements)	Codes
Opportunities for developing knowledge (*n* = 87)	Role development
	Knowledge development
	Developing professional self-assurance
	Supervision

Interacting and building relationships with colleagues and patients (*n* = 81)	Colleagues and coworkers
	Encounter with patients
	Group supervision

Being capable of managing nursing duties (*n* = 27)	Work as a nurse
	Confirmation
	Feels right
	Take responsibility

Experiencing meaningfulness of work (*n* = 21)	To do good
	To contribute

Don't know (*n* = 5)	

**Table 3:** Participant demographics.

Biological sex (*n*)	Male	26
	Female	188
	Other	2

Age	Mean (range)	29.5 (21–52)
	Median	28

Previous working experience in health care (e.g., assistant nurse)	Yes	167
	No	49

Hospitals represented (*n*)		5

**Table 4:** Perceptions of preparedness, satisfaction with choices and future in profession.

	*N*	Mean score (SD)	Agree (*n* (%))
Nursing education has in general prepared me well for working as a nurse	216	3.54 (0.949)	132 (61)
Nursing education has prepared me well for practical work as a nurse	216	3.36 (1.05)	123 (57)
Nursing education has given me relevant theoretical knowledge for working as a nurse	216	3.75 (0.848)	156 (72)
If I were to choose education today, I would choose nursing education again	216	3.96 (1.12)	162 (75)
I enjoy my profession as a nurse	216	4.25 (0.816)	183 (85)
I plan to continue my work as a nurse	216	4.31 (0.890)	181 (84)

Abbreviation: SD = standard deviation.

**Table 5:** Overview of nurses' experience of their working situation on their ward the past week.

	*N*	Mean score (SD)	Agree (*n* (%))
How have you experienced your workload over the past week?	216	3.97 (0.826)	n.a.
I am satisfied with the support I receive in my daily work	216	4.06 (0.876)	170 (79)
The past week, how often have you …			
Felt recovered and rested before starting a new shift?	216	2.80 (1.07)	61 (28)
Felt that you know what responsibilities you have on the healthcare unit?	216	4.24 (0.678)	194 (90)
Felt that you know exactly what is required of you in your work?	216	3.99 (0.747)	173 (80)
Been worried about making mistakes?	216	3.38 (1.18)	96 (44)
Felt that there is someone to ask if you need help?	216	4.35 (0.799)	184 (85)
Felt like a part of the team on the care unit?	216	4.51 (0.753)	195 (90)

## Data Availability

The datasets generated during and analysed during the current study are available from the corresponding author on reasonable request.

## Supporting Information

Completed strengthening the reporting of observational studies in epidemiology (STROBE) checklist for this study.
